# An extended super/subloading surface model for soft rock considering structure degradation

**DOI:** 10.1371/journal.pone.0258813

**Published:** 2021-10-21

**Authors:** Kai Cui, Bin Hu, Aneng Cui, Jing Li, Erjian Wei, Zhen Zhang

**Affiliations:** 1 School of Resources and Environmental Engineering, Wuhan University of Science and Technology, Wuhan, Hubei, China; 2 Hubei Key Laboratory for Efficient Utilization and Agglomeration of Metallurgic Mineral Resources, Wuhan, Hubei, China; China University of Mining and Technology, CHINA

## Abstract

The strain-softening and dilatancy behavior of soft rock is affected by the loading history and the development of structure. This study regards soft rock as a structured and overconsolidated soil and develops a new elastoplastic model based on the classical super yield surface Cam-clay model. The proposed model is capable of capturing the effect of yield surface shape on the mechanical behavior of soft rock by introducing a new yield function. The proposed model is validated against the triaxial test results on different types of soft rocks under drained condition. The comparison results indicate that the proposed model is suitable for describing the constitutive behavior of soft rock.

## 1. Introduction

**Fig 1:**
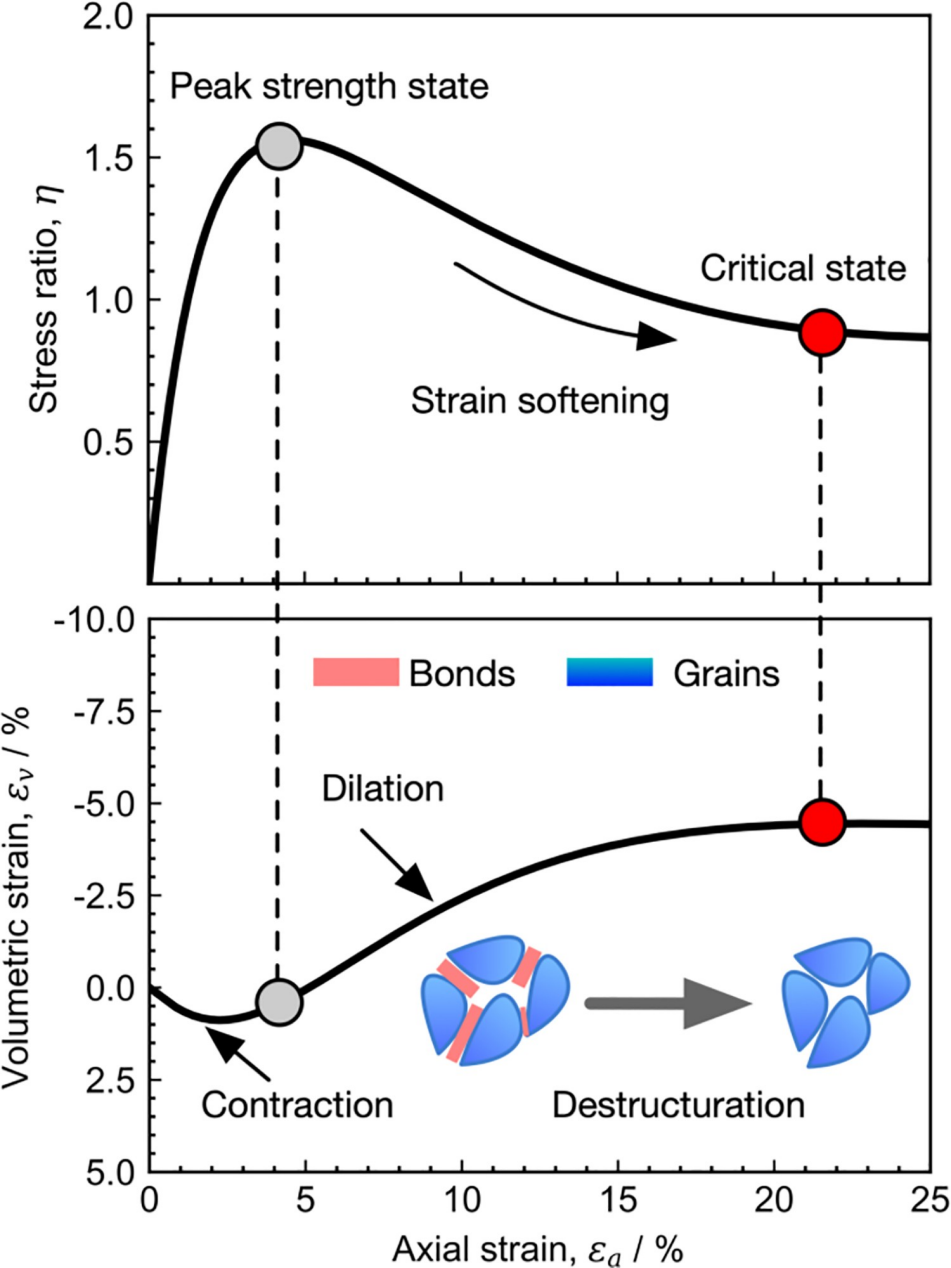
Strain softening and dilatancy features of soft rock.

Understanding the strength and deformation of soft rock is essential for many engineering practice encountered in the mountainous area, such as tunneling excavation [1–4] and slope reinforcement design [5–7]. After saturation, the mechanical behavior of soft rock is similar to that of heavily overconsolidated clay, which exhibits strain-softening and dilatancy features in a drained triaxial test with low confining pressure [8–10], as shown in Fig 1. In addition, soft rock generally develops a complex microstructure during the sedimentation process and further forms a bonded strength [2, 11]. The formation of bonds within particle contacts increases the contact stiffness and hence the macroscopic stiffness [8]. During the deformation process, the internal microstructure in soft rock undergoes irreversible damage. A large number of micro-cracks occur and lead to the reduction of modulus and bond strength. The degradation rate of bonding structure is significantly accelerated with increasing stress [12]. As a result, the residual strength of soft rock will decrease with increased confining pressure.

**Fig 2:**
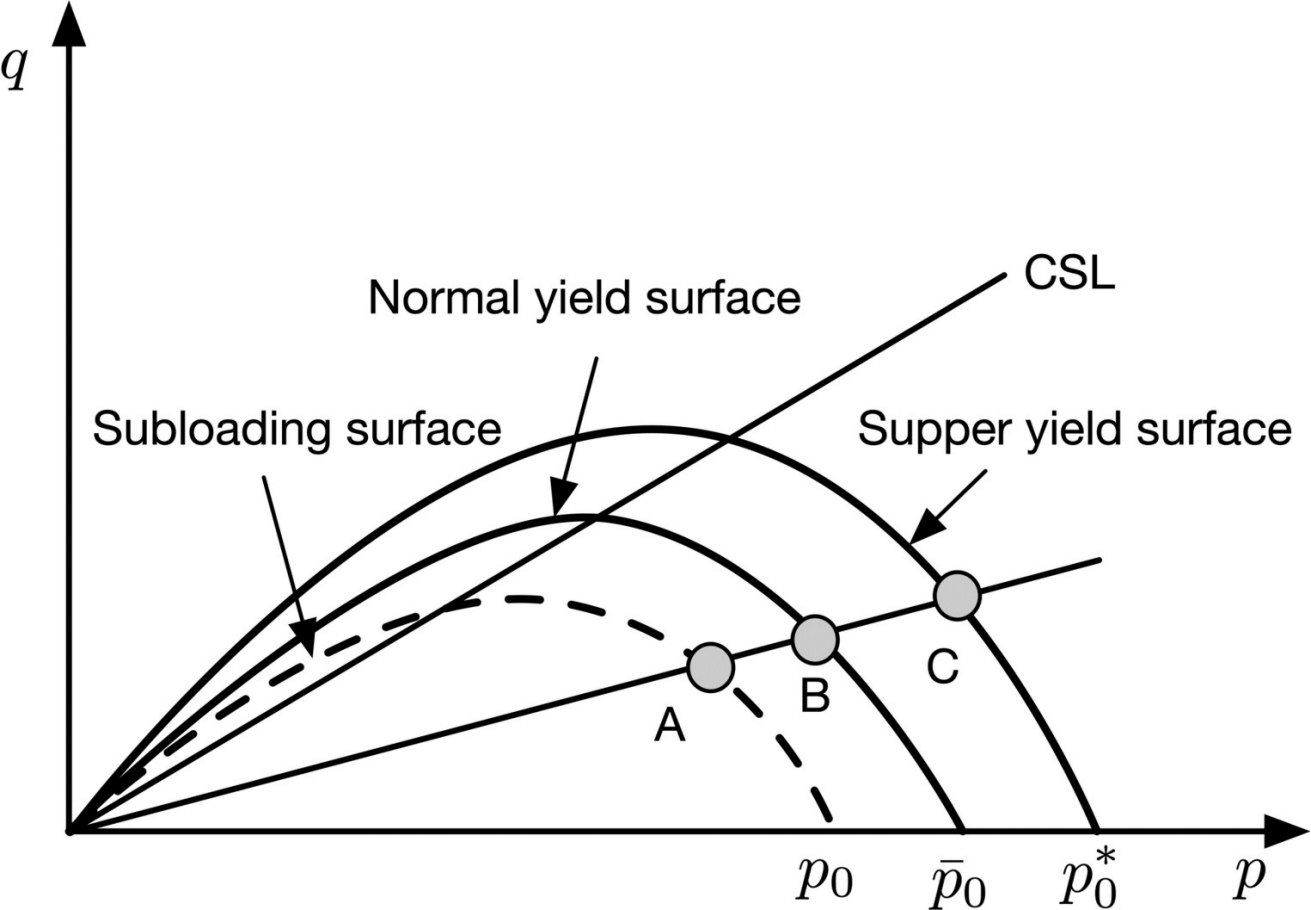
Super yield surface and subloading surface in the p−q plane.

The complicated mechanical behavior of soft rock can be investigated using the framework of critical state soil mechanism since it can be regarded as a structured overconsolidated soil. The super yield surface Cam-clay model (SYS Cam-clay model) developed by Asaoka et al. (2000) [13, 14] can capture the influence of structure and overconsolidation ratio on the deformation characteristics of soils conveniently. As illustrated in Fig 2, the SYS Cam-clay model contains three yield surfaces. Specifically, the normal yield surface represents a normally consolidated state where a reference stress point B remains on it. The subloading surface passes through the current stress point A and is located inside the normal yield surface. The subloading surface concept [15–17] is developed to account for the irreversible plastic deformation at the beginning of shearing and has been proven to be an useful tool in describing soil behaviors. After that, it can be used to capture the overconsolidation effect and provides a smooth transition between the elastic and plastic deformations. In addition, the super yield surface is beyond the normal yield surface with larger isotropic yield stress. The size of the super-loading surface is controlled by the current degree of structure.

Numerous constitutive models have been developed in the literature based on the SYS Cam-clay model for structured soil, including frozen soil [18], cemented soil [19], and soft rock [8]. However, those models cannot consider the effect of shape of yield surface on the mechanical behavior of soft rocks. Thereafter, it is reasonable to suggest that the performance of the SYS Cam-clay model can be improved by employing a flexible yield function for describing various shapes of yield surface of structured soil.

Similar to the SYS Cam-clay model, the constitutive model for clay and sand developed by Yu (1998) [20], referred to as CASM, is also widely applied in literature due to its simplicity and flexibility in describing the shape of the yield surface [21–25]. More importantly, the concept of spacing ratio can represent the relation between critical state point and yield surface stress point, which is regarded as an essential parameter in describing soil behavior. Normally, the value of the spacing ratio for different types of soils is not constant. For example, the spacing ratio of intermediate-graded sand [26] depends on the initial density. Besides, according to Kang and Liao (2020) [27], the spacing ratio for jointed soft rock is affected by the orientation angle of the joint plane. In this study, the spacing ratio concept is employed into the SYS Cam-clay model to consider the effect of shapes of the yield surface on model predictions.

This paper aims to develop a new elastoplastic constitutive model for soft rock based on the SYS Cam-clay model. In the proposed model, the effects of structure and overconsolidation ratio are captured by the super yield surface and subloading surface, respectively. The related yield functions are given by the CASM model to consider the effect of shape of yield surface on the deformation characteristics of soft rock additionally. The proposed model is validated against the triaxial test data of different types of soft rocks under drained and undrained conditions.

## 2. Model formulation

In this study, the model formulation is carried out using the notations appropriate for triaxial tests. Compressive stresses and contrastive strains are assumed to be positive following the geotechnical conventions. In this section, the principles of effective stress and critical state soil mechanics are used to develop the elastoplastic model for soft rock. Correspondingly, the mean effective stress *p* and the shear stress *q* are respectively defined as $p=\frac{\sigma_{1}+2\sigma_{3}}{3}$ and *q* = *σ*_1_−*σ*_3_, where *σ*_1_ and *σ*_3_ denote effective vertical and radial stresses, respectively. The corresponding strains are defined as *ε*_*v*_ = *ε*_1_+2*ε*_3_ and $\varepsilon_{s}=\frac{2}{3}(\varepsilon_{1}-\varepsilon_{3})$, where *ε*_1_ and *ε*_3_ denote vertical and radial strains, respectively.

### 2.1 The state-stress relation and yield function

**Fig 3:**
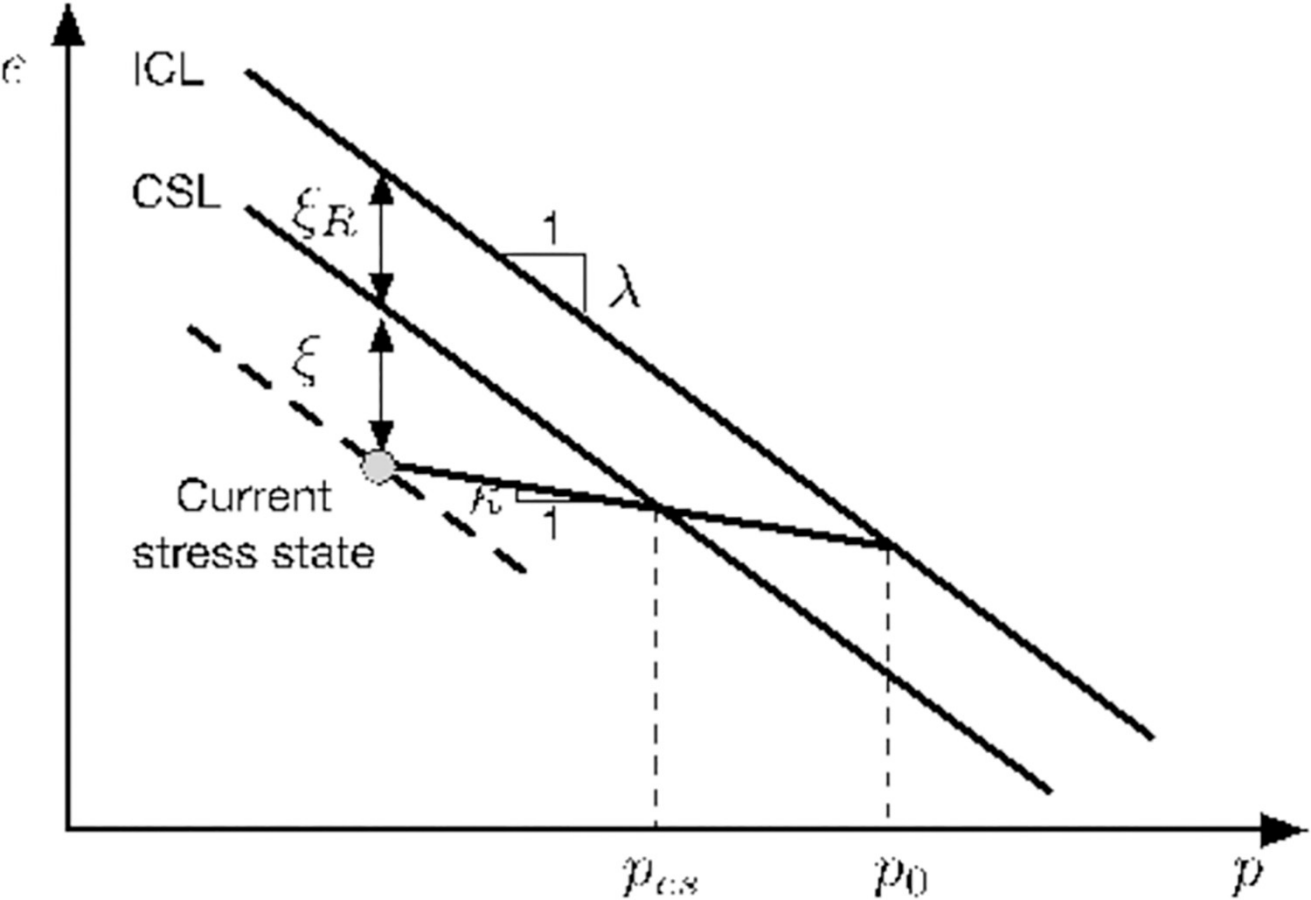
State parameters in the e−ln p plane.

Fig 3 shows the isotropic consolidation line (ICL) and critical state line (CSL) in the stress versus void space. The vertical distance between the ICL and CSL is represented by a reference state parameter *ξ*_*R*_ defined as *ξ*_R_ = (*λ*−*κ*)ln*r*, where *r* is the spacing ratio, and *λ* and *κ* are the slopes of the ICL and a loading/unloading curve, respectively. The spacing ratio is defended as the ratio of preconsolidated pressure to mean normal stress corresponding to the critical state. For the original Cam-clay (CC) and modified Cam-clay (MCC) models [28], the values of *r* are predefined as 2.7 and 2.0, respectively. However, experimental observations indicate that *r* has a specific value for different types of soils. According to Yu (1998) [20], *r* typically ranges between 1.5–3.0 for clays, but for sands, the value of *r* is generally much larger.

The relative position between the current stress point and the CSL is determined by the state parameter *ξ* developed by Been and Griffies (1978) [29]. *ξ* is defined as the difference between the current void ratio *e* and the critical state void ratio *e*_*cs*_ with a given mean effective pressure. In the CASM model, *ξ* controls the material behavior before the achievement of the critical state and can be mathematically expressed as:

$$\xi =e+\lambda \ln(p/p_{r})-\Gamma$$ (1)

where *Γ* is a reference void ratio on the CSL when the reference pressure *p*_*r*_ = 1 kPa.

The state boundary surface in the CASM model for a variety of soils is represented with the following general stress-state relation:

$${\left(\frac{\eta }{M}\right)}^{n}=1-\frac{\xi }{\xi_{R}}$$ (2)

where *η* is the stress ratio defined as *η* = *q*/*p*; *M* is the slope of the CSL in the *p*−*q* plane; and *n* is a material constant with a typical value ranging from 1 to 5 [30].

The general stress-state relation leads to a new yield function in terms of the preconsolidated pressure *p*_0_ as follows:

$$f=\ln\frac{p}{p_{0}}+\ln r{\left(\frac{\eta }{M}\right)}^{n}=0$$ (3)

The derived yield function allows the CASM model to predict local peaks in the deviatoric stress on the left side of the critical state condition because the intersection between the CSL and the yield surface may occur before reaching the critical state. In addition, the yield function can predict a vast majority of shapes of the yield surface.

**Fig 4:**
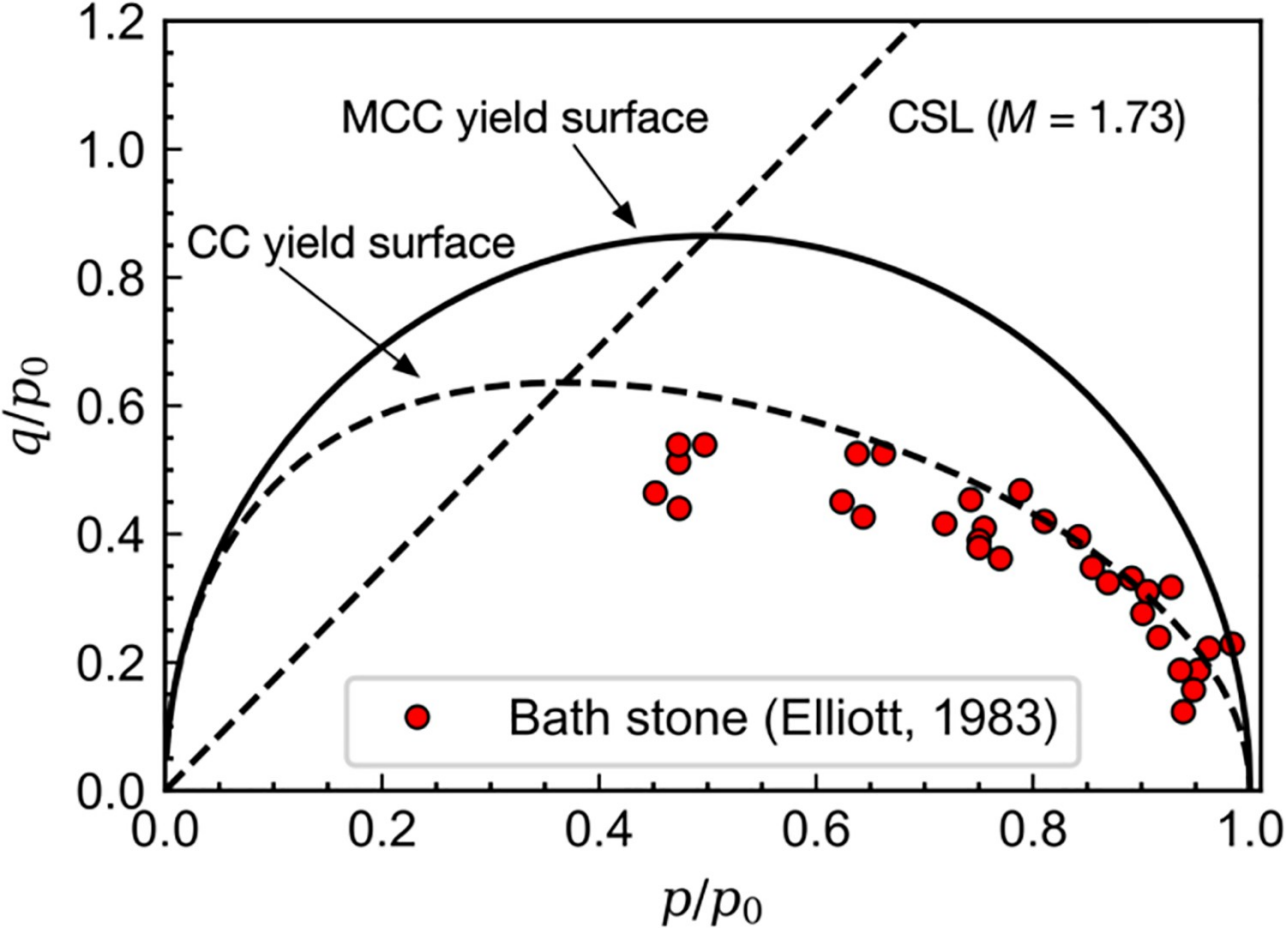
Measured and calculated yield surface of Bath stone.

**Fig 5:**
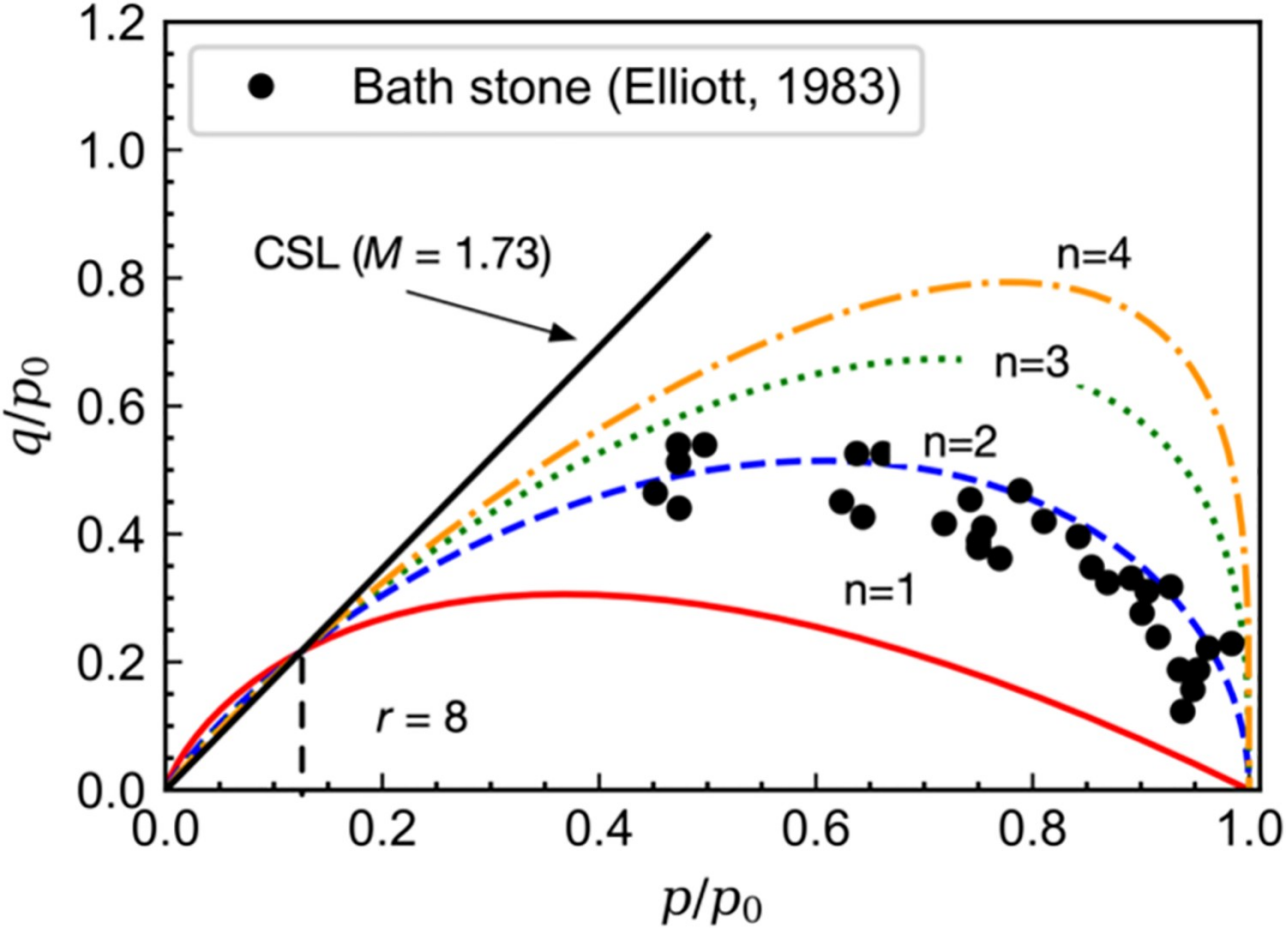
Effect of parameter r on the shape of the yield surface.

Fig 4 shows the experimental yield surface of Bath stone [31]. It can be found that the yield function of the modified Cam-clay model cannot capture the measured yield points. The prediction by that of the original Cam-clay model seems to be better, but a slight discrepancy still exists. However, the yield surface given by Eq (3) can fit well with the experimental data of Bath stone on the ductile side when *n* = 1.7 and *r* = 8, as shown in Fig 5. In this case, the strain vector at the intersection point between the yield surface and the critical state line is not normal to the yield surface. Therefore, a non-associated flow rule may be suggested to ensure no additional volumetric deformation occurred at the critical state. It also indicates that the yield surface will be extended without changing the intersection location with increasing value of *n*.

### 2.2 Yield surfaces and plastic deformation

**Fig 6:**
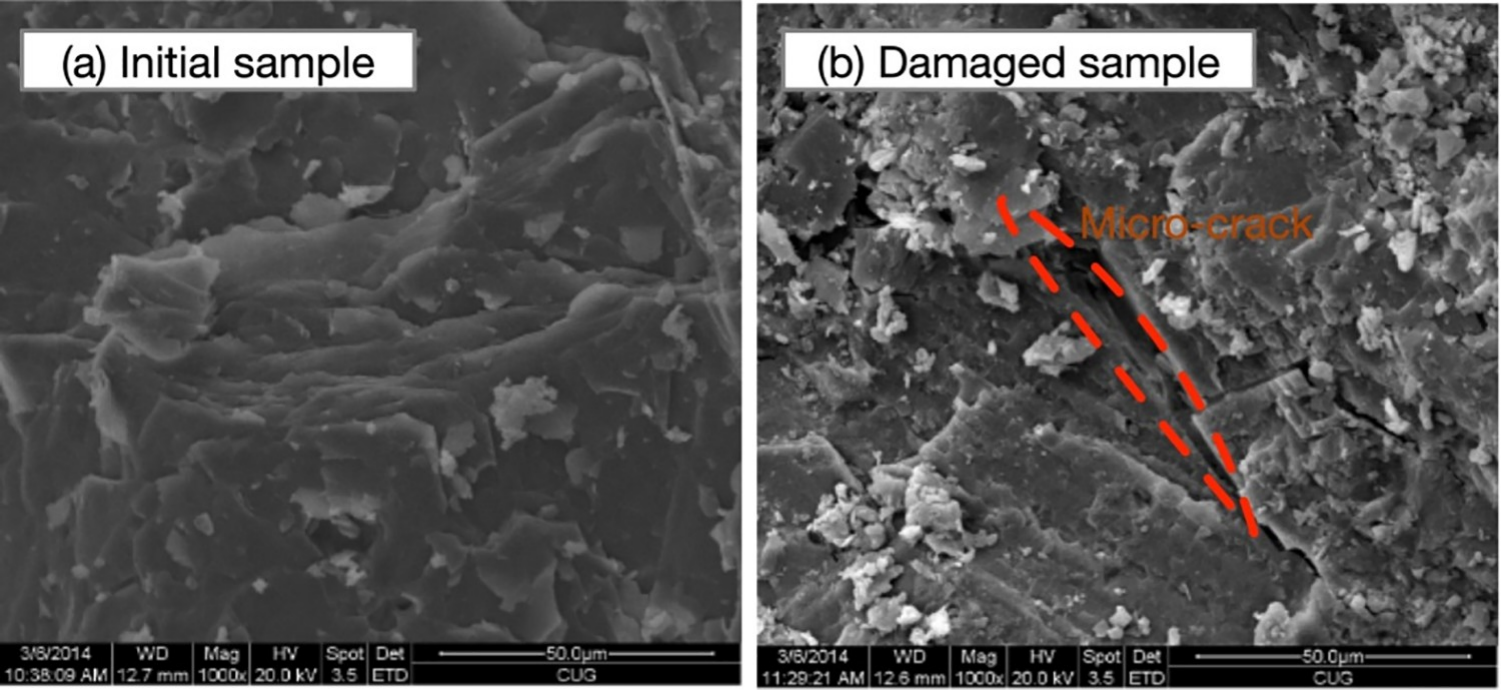
SEM images of Carbonaceous mud shale.

The soft rock may exhibit dilatancy and strain-softening features due to significant structure and overconsolidation effects. During a loading process, the bond strength will be gradually decreased with the development of micro-cracks inside the soft rock, as shown in Fig 6, which is obtained using the scanning electron microscopy (SEM) imaging approach for Carbonaceous mud shale.

**Fig 7:**
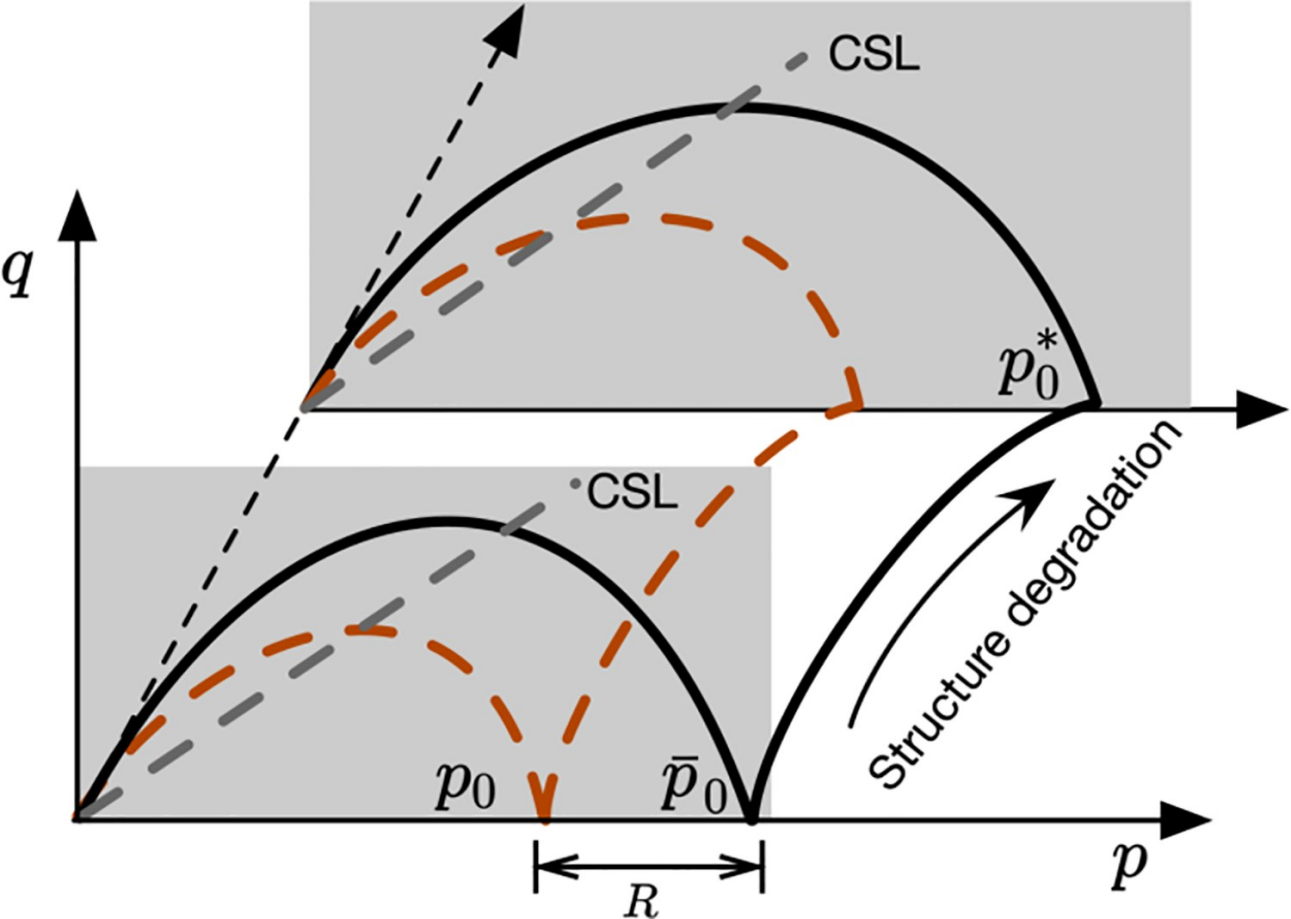
Definitions of overconsolidation parameter R and structuration parameter R*.

In this study, the concept of super yield surface and subloading surface are employed to capture the structure and overconsolidation effects. Fig 7 is the schematic diagram that indicates the change in size of those yield surfaces during shearing. The structure degradation is mathematically expressed by the newly defined structuration parameter R*. Meanwhile, the dissipation of the overconsolidation is controlled by the overconsolidation *R*. The definitions of R* and *R* are given by:

$$R=\frac{p}{\bar{p}}=\frac{q}{\bar{q}},\;R^{*}=\frac{\bar{p}}{p^{*}}=\frac{\bar{q}}{q^{*}}$$ (4)

The soft rock will return to a normally consolidated state when *R* = 1. In addition, the structure of soft rock will be completely damaged when R* = 1. Based on the definitions of parameters *R* and R*, the subloading surface can be expressed as:

$$f=\ln\frac{p}{p_{x0}}+\ln r{\left(\frac{\eta }{M}\right)}^{n}+\ln R^{*}-\ln R-\frac{1+e_{0}}{\lambda -\kappa }d\varepsilon_{v}^{p}=0$$ (5)

where *p*_*x*0_ is the initial mean effective stress and *e*_0_ is the initial void ratio.

By taking total derivative to Eq (5), the consistency condition can be obtained as:

$$df=\frac{\partial f}{\partial p}dp+\frac{\partial f}{\partial a}dq+\frac{\partial f}{\partial R^{*}}dR^{*}+\frac{\partial f}{\partial R}dR+\frac{\partial f}{\partial \varepsilon_{v}^{p}}d\varepsilon_{v}^{p}=0$$ (6)

The derivatives of yield function *f* with respect to the elements in Eq (5) are derived as:

$$\frac{\partial f}{\partial p}=\frac{1}{p}\left[1-n\ln r{\left(\frac{\eta }{M}\right)}^{n}\right]$$ (7A)

$$\frac{\partial f}{\partial q}=\frac{n\ln r}{q}{\left(\frac{\eta }{M_{cs}}\right)}^{n}$$ (7B)

$$\frac{\partial f}{\partial R^{*}}=\frac{1}{R^{*}}$$ (7C)

$$\frac{\partial f}{\partial R}=-\frac{1}{R}$$ (7D)

$$\frac{\partial f}{\partial \varepsilon_{v}^{p}}=-\frac{1+e_{0}}{\lambda -\kappa }$$ (7E)

The evolution laws for the parameters *R* and R* used in the study are as the same as Asoka et al. (2000) [13, 14]:

$$dR=-\frac{1+e_{0}}{\lambda -\kappa }\frac{aM\ln R}{R}d\varepsilon_{s}^{p}$$ (8)

$$dR^{*}=\frac{1+e_{0}}{\lambda -\kappa }MR^{*}(1-R^{*b})d\varepsilon_{s}^{p}$$ (9)

where *a* and *b* are material constants that control the dissipation rate of structure and overconsolidation, respectively.

### 2.3 Plastic potential and elastic deformation

According to the conventional plastic theory, the definition of strain is given by:

$$d\epsilon =d\epsilon^{e}+d\epsilon^{p}=d\epsilon^{e}+\Lambda \frac{\partial g}{\partial \sigma }$$ (10)

where superscripts ‘e’ and ‘p’ designate the elastic and plastic components, respectively; and *g* is the plastic potential function. The proposed mode uses a non-associated flow rule that follows the Rowe’s stress-dilatancy law [32], which has been applied with success when describing the deformation of geomaterials.


$$\frac{d\varepsilon_{v}^{p}}{d\varepsilon_{s}^{p}}=\frac{9(M-\eta )}{9+3M-2M\eta }$$ (11)

By integrating Eq (11), the plastic potential function of the proposed model is obtained as:

$$g=3M\ln\left(\frac{p}{\varphi }\right)+(3+2M)\ln\left(\frac{2q}{p}+3\right)+\left(M-3\right)\ln\left(3-\frac{q}{p}\right)=0$$ (12)

where *φ* is a size parameter controlling the size of the plastic potential which passes through the current stress state. It indicates that Eq (12) does not depend on the hardening parameters.

Based on the generalized Hooke’s Law, the elastic deformation of soft rock can be calculated using the effective modulus. The corresponding bulk modulus *K* and shear modulus *G* can be expressed by the elastic modulus *E* and Poisson’s ratio *v* as [28]:

$$K=\frac{E}{3(1-2v)},\;G=\frac{E}{2(1+v)}$$ (13)

Thereafter, the elastic volume strain $\varepsilon_{v}^{e}$ and elastic shear strain $\varepsilon_{s}^{e}$ of soft rock are:

$$\varepsilon_{v}^{e}=\frac{p}{K},\;\varepsilon_{s}^{e}=\frac{q}{3G}$$ (14)

### 2.4 Incremental stress-strain relationship

The conventional solution of the incremental stress-strain relationship is based on explicit integration of the following rate constitutive equation:

$${\dot{\sigma }}_{ij}=[C_{ijkl}^{e}-\langle C_{ijkl}^{p}\rangle ]{\dot{\varepsilon }}_{ij}$$ (15)

where $\dot{\sigma }$ and $\dot{\varepsilon }$ are the incremental stress and strain tensors, respectively. The symbols ⟨⋅⟩ is the macauley brackets which implies that ⟨*x*⟩ = *x* when plasticity is in effect; otherwise, ⟨*x*⟩ = 0.

The elastic stress-strain tensor $C_{ijkl}^{e}$ is given by:

$$C_{ijkl}^{e}=K\delta_{ij}\delta_{kl}+G(\delta_{ik}\delta_{jl}+\delta_{il}\delta_{jk})$$ (16)

where *δ*_*ij*_ is the Kronecker delta.

The plastic stress-strain tensor $C_{ijkl}^{p}$ is given by:

$$C_{ijmn}^{p}=\frac{C_{ijmn}^{e}\frac{\partial g}{\partial \sigma_{mn}}\frac{\partial f}{\partial \sigma_{st}}C_{stkl}^{e}}{H+\frac{\partial f}{\partial \sigma_{ij}}C_{ijkl}^{e}\frac{\partial g}{\partial \sigma_{kl}}}$$ (17)

where *H* is the plastic modulus with the following definition:

$$H=-\frac{\partial f}{\partial q_{n}}\frac{\partial q_{n}}{\partial \varepsilon_{v}^{p}}\frac{\partial g}{\partial \sigma_{ii}}\frac{\delta_{ij}}{3}$$ (18)

### 2.5 Model parameters

The proposed model contains three groups of parameters that can be determined through conventional laboratory tests. Firstly, the elastoplastic parameters from the modified Cam-clay model, including compression index *λ*, recompression index *κ*, initial void ratio *e*_0_, critical state line slope *M*, and Poisson’s ratio *v*. Secondly, the structure and overconsolidated parameters R* and *R*, the corresponding evolution parameters *a* and *b*. Thirdly, the spacing ratio *r* and parameter *n* for the stress-state relation, which are additional parameters compared with the super/subloading yield surfaces Cam-clay model. By changing the values of *r* and *n*, the shapes of yield surface can be adjusted to fit the measured yield points better and improve model performance.

## 3. Model validation

The proposed model is validated using drained triaxial test results on different types of soft rocks, including Carbonaceous mud shale [33], Ohya rock [34] and Colorado shale [35, 36]. The corresponding material parameters are calibrated in Table 1.

**Table 1:** Model constants and initial values of the internal variables for the Carbonaceous mud shale, Ohya rock and Colorado shale.

Parameter	Carbonaceous mud shale [33]	Ohya rock [34]	Colorado shale [35, 36]
*e*_0_	0.38	1.08	0.72
*M*	2.72	1.3	2.1
*λ*	0.0165	0.0242	0.0122
*κ*	0.0021	0.0092	0.0034
*v*	0.25	0.25	0.25
*Γ*	0.46	1.25	0.82
*r*	2.1	2.0	1.9
*n*	1.5	1.4	1.4
*a*	0.472	0.72	0.56
*b*	0.1	0.2	0.16
*R*_0_	0.12	0.26	0.34
$R_{0}^{*}$	0.92	0.85	0.88

### 3.1 Carbonaceous mud shale

The capability of the proposed model in describing the behavior of soft rock is firstly validated using the triaxial test results on carbonaceous mud shale [33] under the drained condition. During the test, the confining pressures are 100, 200, 300, and 400 kPa, respectively. The preconsolidated pressure is assumed to be 50 MPa to determine the initial value of the overconsolidation parameter *R*. The structuration parameter R* is independent of the confining pressure, whose initial value is assumed to be 0.6 herein. The values of the material parameters in the proposed model are given in Table 1.

**Fig 8:**
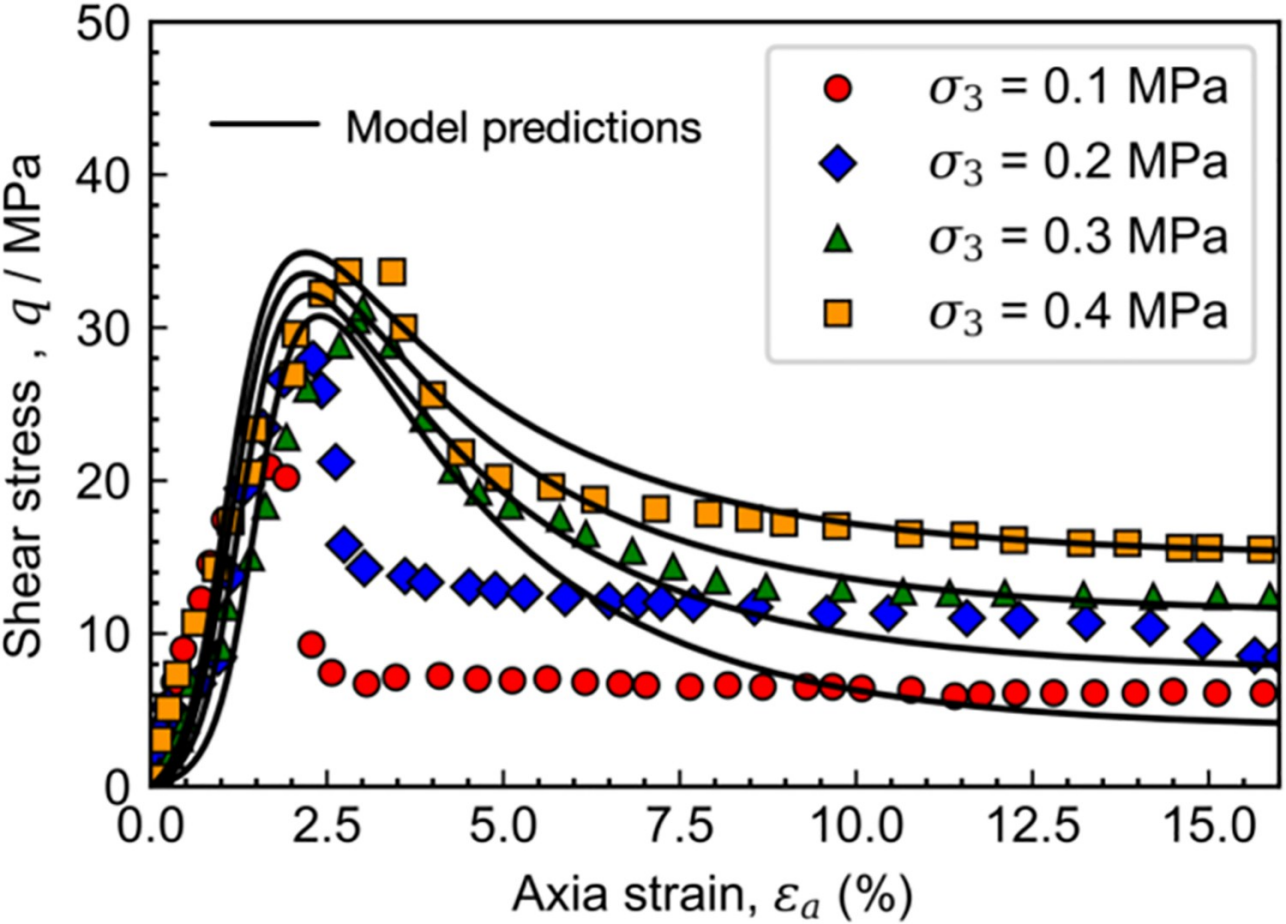
Measured and calculated stress-strain relations of Carbonaceous mud shale.

Fig 8 shows the measured and calculated stress-strain relations of carbonaceous mud shale. It can be found that the model predictions are in agreement with the experimental data, and the strain-softening feature of stress-strain relations is reasonably captured. However, it should be noted that the predicted peak strength at a lower stress level is much smaller than the measured value. For example, in the cases of the confining pressure equals to 100 and 200 kPa, the measured peak strength is approximately 2900 and 2200 kPa, respectively. The maximum discrepancy between the measured value and model predictions is thus more than 20%. One of possible reasons for this discrepancy is that the structuration effect on the critical state line is not considered in the proposed model [34], which is beyond the scope of the current study.

**Fig 9:**
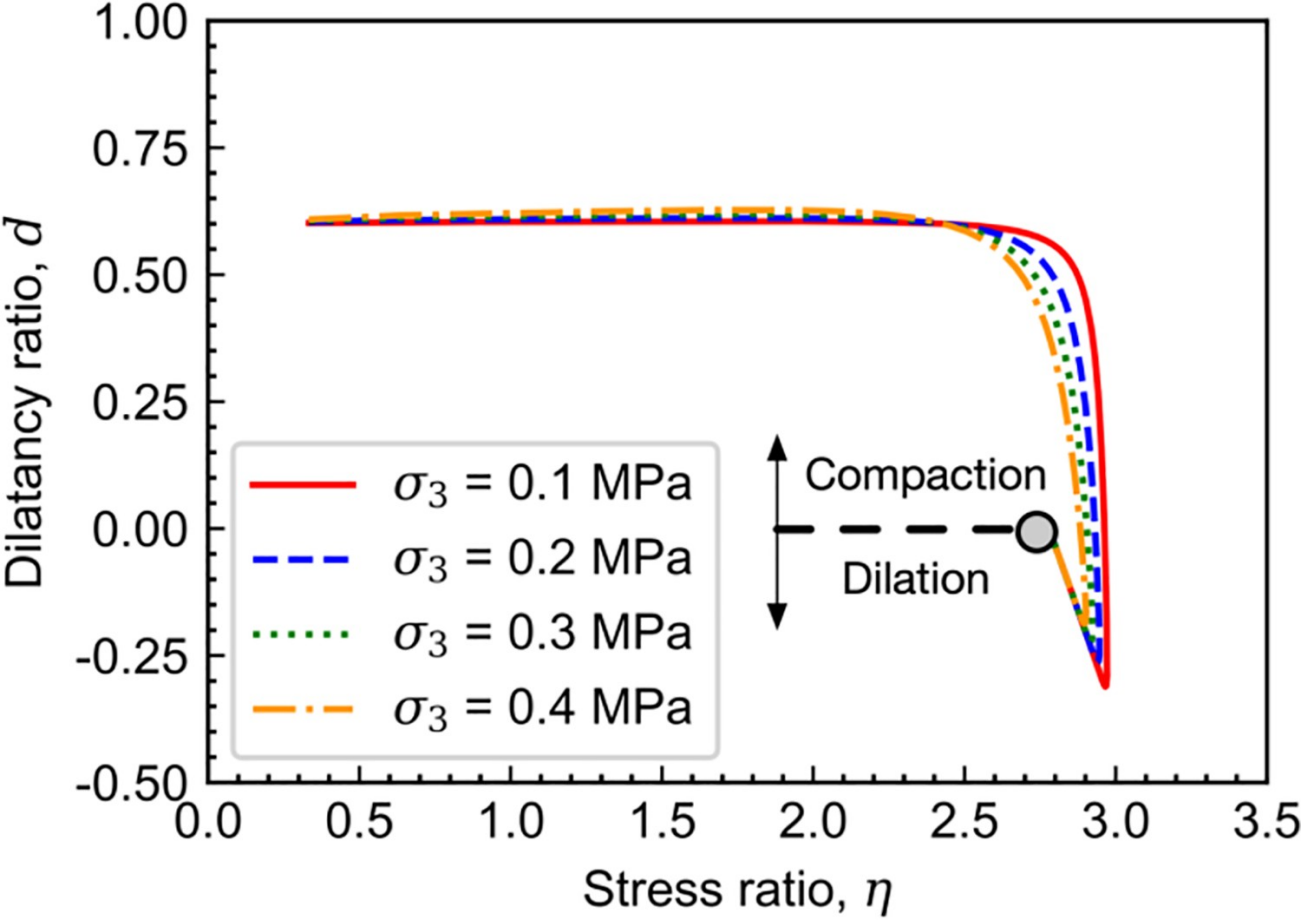
Calculated stress-dilatancy relations of Carbonaceous mud shale.

Fig 9 shows the calculated stress-dilatancy relations of carbonaceous mud shale. It can be found that at the beginning of shearing, the dilatancy ratio *d* decreases with the increased stress ratio *η*. When the current stress point reaches the phase transformation line where the incremental volumetric strain equals to zero, further loading will lead *d* to be negative, and dilatancy in volumetric deformation can be observed consequently. After the peak strength state, the current stress point will move backward to the final critical state. Thereafter, both stain-softening and dilatancy features of soft rock can be captured by the proposed model.

### 3.2 Ohya rock

The triaxial test data of Ohay rock under the drained condition was reported by Fu et al. (2016) [34]. The cylindrical rock samples with 50 mm in diameter and 100 mm in height were mined at Ohya Village of Tochigi Prefecture. Before shearing, the samples were first isotropic consolidated for 24 hours under the prescribed confining pressure (*σ*_3_ = 0.5 MPa). Then, the shearing was conducted in 0.001%/min under drained conditions with the confining pressure of 0.5, 1.0, 1.5, and 2.0 MPa.

**Fig 10:**
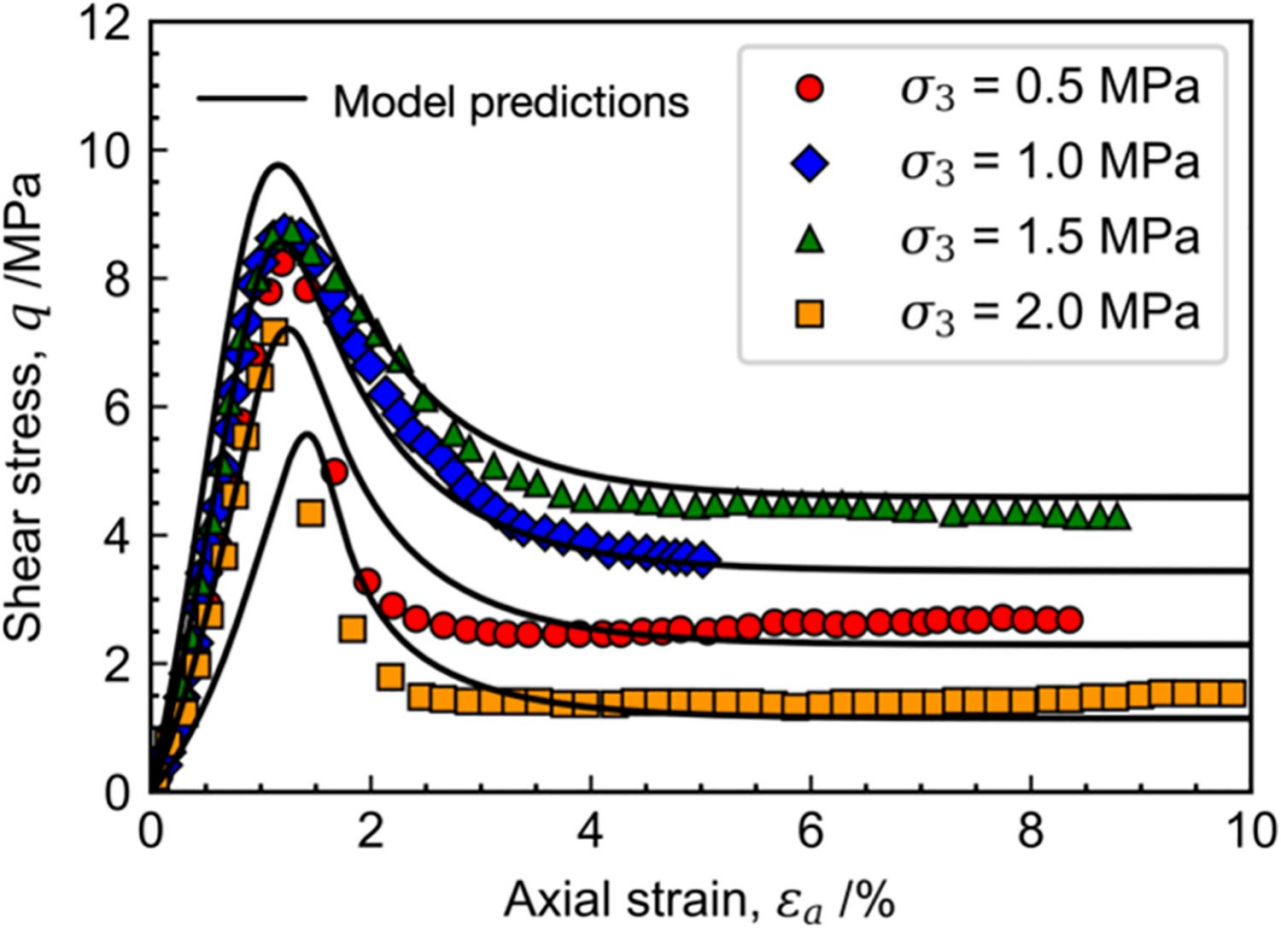
Measured and calculated stress-strain relations of Ohay rock.

**Fig 11:**
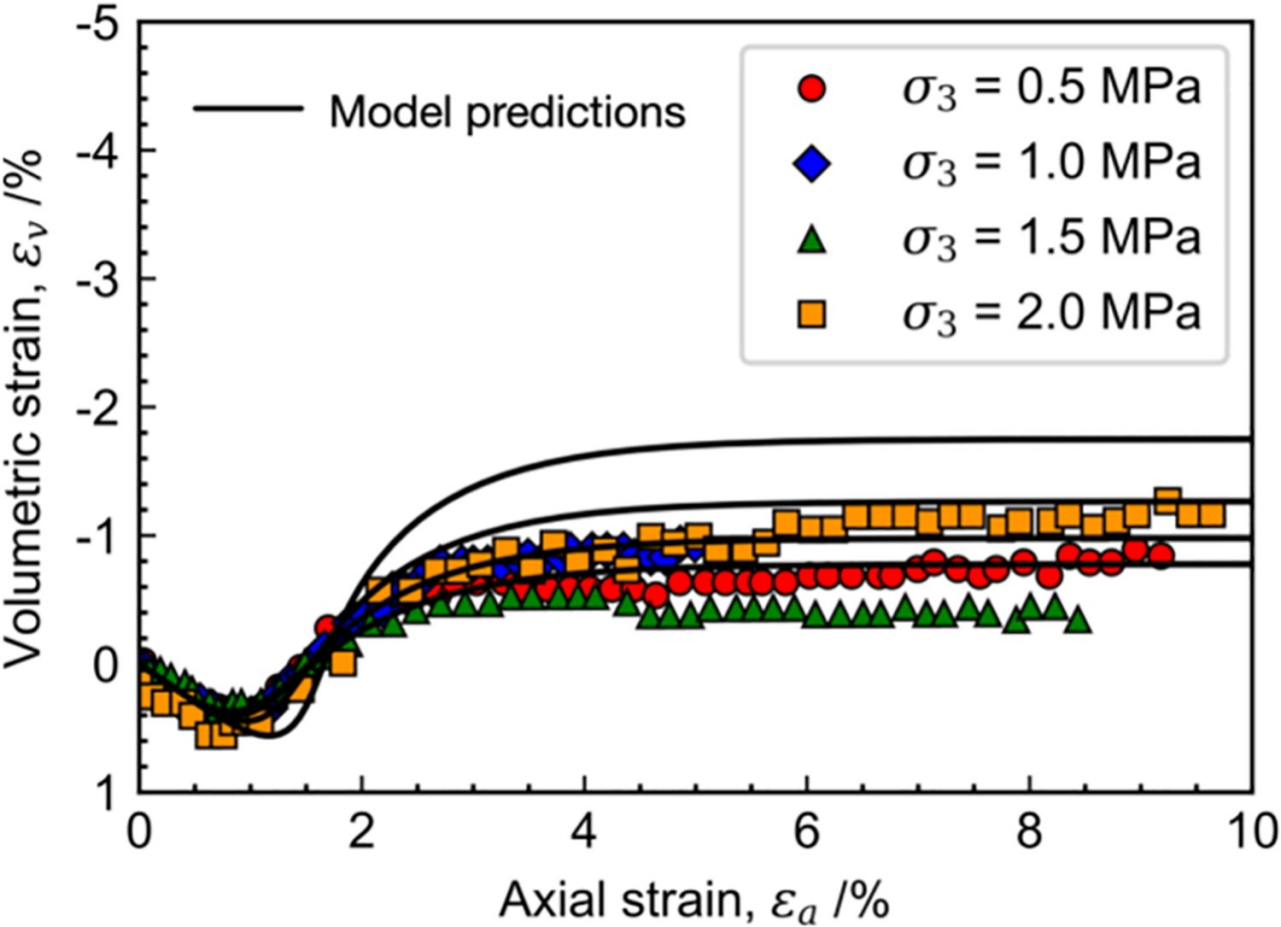
Measured and calculated volumetric deformation of Ohay rock.

Fig 10 shows the measured and calculated stress-strain relations of Ohay rock, the calibrated material parameters in the proposed model are given in Table 1. It can be found that the rock samples exhibit strain-softening behavior under all test conditions. With the increment of confining pressure, the tangent modulus and peak strength are increased in various degrees. The model predictions agree with the measured stress-strain relations, especially after the post-peak strength state. The results of volumetric strain versus axial strain are illustrated in Fig 11. It can be found that the rock samples are compacted at the early loading stage with a positive incremental volumetric strain. During that process, only a few micro-creaks occur of the rock samples, and the mechanical behavior of rock is mainly affected by the dissipation of overconsolidation. After that, many micro-cracks are developed and connected to form large cracks due to shearing. As a result, the strength of rock samples is significantly reduced, and the volumetric deformation turns to be expansive. Note that the predictions of final volumetric strain at different confining pressures are larger than the measured values with the maximum discrepancy of 12%. The performance of the proposed model can be improved by slightly adjusting the shapes of the yield surface, and more laboratory tests are needed to be conducted in this case to determine the exact values of parameters *r* and *n*.

### 3.3 Colorado shale

**Fig 12:**
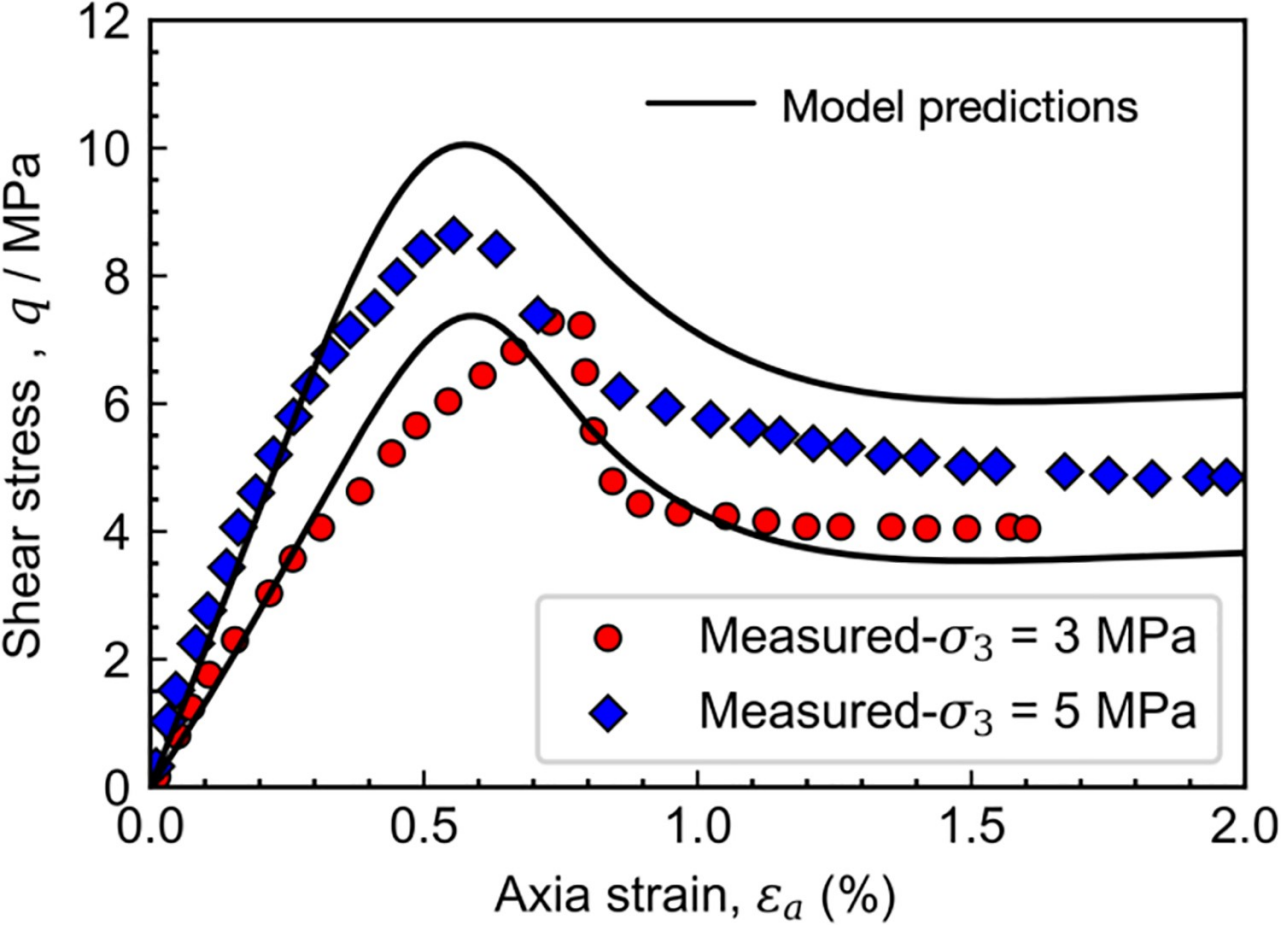
Measured and calculated stress-strain relations of Colorado shale.

**Fig 13:**
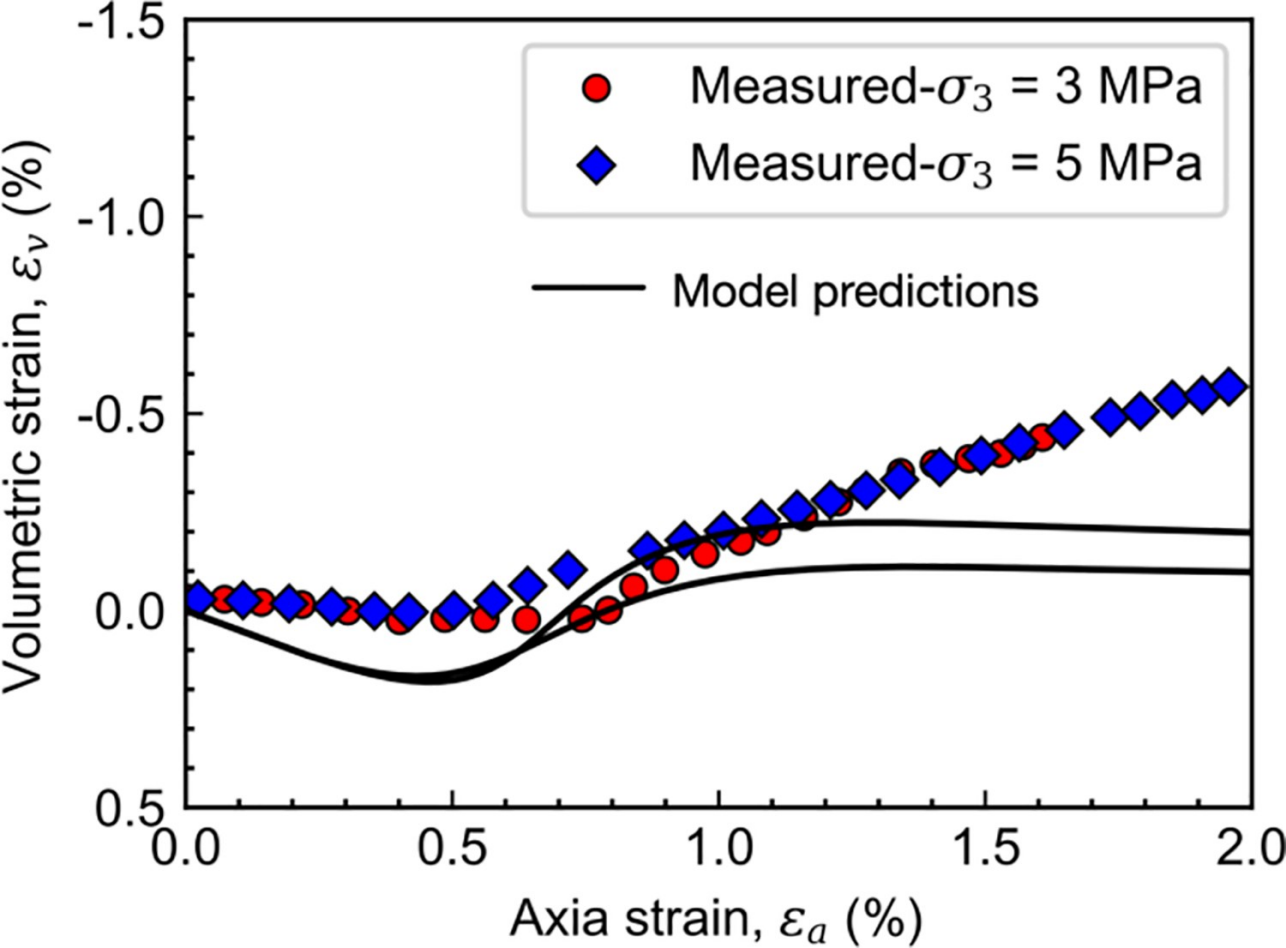
Measured and calculated volumetric deformations of Colorado shale.

Figs 12 and 13 shows the comparison between model predictions and drained triaxial test results on [35, 36], the calibrated material parameter in the proposed model is given in Table 1. It is noteworthy that the quality of the match between calculated and measured stress-strain-volumetric responses deteriorates in the post-peak regime. For example, the predicted peak strengths are approximately 11.2 and 7.2 MPa when the confining pressures are 3 and 5 MPa, respectively. However, the corresponding measured values are 8.6 and 6.6 MPa. At the same time, the predicted final dilatancy is much smaller than the measured ones. Those deteriorations may be attributed to the occurrence of shearing bands due to localized deformation. Although there are some discrepancies in the value of peak strength and final dilatancy between the model predictions and test results, the primary behavior of Colorado shale, including strain-softening and dilatancy, are well described by the proposed model. Besides, the effects of overconsolidation and structure degradation on behavior of rock are considered herein.

## 4. Conclusions

This study regards soft rock as a structured and overconsolidated soil and develops a new elastoplastic model based on the classic super yield surface Cam-clay model. The proposed model can additionally account for the shape effect of the yield surface on constitutive behavior of soft rock by introducing a new yield function. The proposed model is validated by against the triaxial test results on different types of soft rocks under the drained and undrained conditions. The following conclusions are obtained:

1. Mechanical behavior of soft rock such as strain-softening and dilatancy is affected by the overconsolidation and structure. Those effects can be considered by employing the super yield surface and subloading surface concepts in the conventional plastic theory;
2. The proposed model uses a new yield function to represent the super yield surface and the subloading surface. The yield function can flexibly describe the various shapes of the yield surface and then improves the model performance in capturing the strength and deformation characteristics of soft rock;
3. The material parameters in the proposed model have clear physical meanings and can be calibrated using laboratory tests. The calculation results by the proposed model are in agreement with the test results of different types of soft rocks. It indicates that the proposed model is capable of accounting for the strain-softening and dilatancy features of soft rock.
